# Combination of t(4;14), del(17p13), del(1p32) and 1q21 gain FISH probes identifies clonal heterogeneity and enhances the detection of adverse cytogenetic profiles in 233 newly diagnosed multiple myeloma

**DOI:** 10.1186/s13039-017-0327-3

**Published:** 2017-07-01

**Authors:** Thomas Smol, Annika Dufour, Sabine Tricot, Mathieu Wemeau, Laure Stalnikiewicz, Franck Bernardi, Christine Terré, Benoît Ducourneau, Hervé Bisiau, Agnès Daudignon

**Affiliations:** 1 Service d’Hématologie-Immunologie-Cytogénétique, CH Valenciennes, Valenciennes, France; 2 Université de Lille Nord de France, Lille, France; 3 Laboratoire de Cytogénétique Hématologique, CH Versailles, Le Chesnay, France; 4 Service d’Hématologie Clinique, CH Valenciennes, Valenciennes, France; 5 Service d’Hématologie Clinique, CH Arras, Arras, France; 6 Service d’Hématologie Clinique, CH Lens, Lens, France; 7 Laboratoire de Biologie, CH Douai, Douai, France; 8 Institut de génétique médicale, Hôpital Jeanne de Flandre, CHRU Lille, Lille, France

**Keywords:** Multiple Myeloma, Interphase FISH, 1q21 gain, 1p32 deletion, Thresholds

## Abstract

**Background:**

Our aim was to set the FISH combination of del(17p13), t(4;14), 1q21 gain and del(1p32), four adverse cytogenetic factors rarely evaluated together, and compare our technical thresholds with those defined in the literature.

**Methods:**

Two hundred thirty-three patients with MM at diagnosis were studied using FISH to target 4 unfavorable cytogenetic abnormalities: 17p13 deletion, t(4;14) translocation, 1p32 deletion and 1q21 gain. Technical thresholds were determined for each probe using isolated CD138-expressing PC from patients without MM.

**Results:**

The FISH analysis identified abnormalities in 79.0% of patients. Del(17p13) was detected in 15.0% of cases, t(4;14) in 11.5%, 1q21 gain in 37.8% and del(1p32) in 8.7%. Adding 1p32/1q21 FISH probes has enabled us to identify adverse cytogenetic profiles in 39.0% of patients without del(17p13) or t(4;14). Clonal heterogeneity was observed in 51.1% of patients as well as an increase in the number of adverse abnormalities when related clones were greater than or equal to 2 (85.1% against 45.6%).

**Conclusion:**

FISH allowed detecting accumulation of adverse abnormalities and clonal heterogeneity in MM with a combination of 4 probes. The impacts of these two parameters need to be evaluated, and could be included in future cytogenetic classifications.

## Background

Multiple Myeloma (MM) is a heterogeneous disease characterized by the clonal proliferation of abnormal plasma cells (PC), invading mainly the bone marrow (BM). MM accounts for approximately 1% of all cancers, and 10% of all hematopoietic neoplasms with a median age of 70 years at diagnosis [1]. The diagnosis of MM is based on BM infiltration by 10% or more clonal PC or dystrophic PC together with evidence of end organ damage [1].

Cytogenetic analysis plays a major role in the risk stratification of MM [2–4]. However, with a metaphase cytogenetic approach, only 35% of patients present abnormal karyotypes, often associated with an advanced stage of the disease [5]. Thus, practice guidelines now recommend interphase fluorescence in-situ hybridization (FISH) as the initial cytogenetic analysis for MM [6]. FISH is performed on isolated CD138-expressing plasma cells [7]. PC enrichment provides a pure tumour population that enables abnormalities to be detected, irrespective of proliferation and infiltration index.

The efficiency of the cell enrichment method limits the number of targets investigated by FISH and a selection of relevant probes is required to provide information on the diagnosis and prognosis. Many combinations of cytogenetic markers have been evaluated. Routine panels mainly evaluate the deletion of 17p13 (*TP53* deletion) and the t(4;14)(p16;q32) *FGFR3-IGH* translocation but do not cover the heterogeneity of MM. The del(17p13) remains the most powerful cytogenetic factor regardless of the therapeutic choice, while t(4;14) loses its negative impact with proteasome inhibitors regimens [8]. Other rearrangements involving *IGH* genes have been reported, such as t(14;16)(q32;q23) and t(14;20)(q32;q12). The prognostic values of the latter vary depending on working groups or methods used [9–11]. In contrast, partial aneuploidies of chromosome 1 (1q21 gain and 1p32 deletion) are retained as more relevant additional markers [10, 12], even with the emerging therapeutic approaches [13, 14]. The addition of 1q21 gain probe is beginning to be integrated into the FISH panel, whereas the identification of 1p32 deletion is not widely used. Moreover, studies are focused on the presence or the absence of FISH markers without considering the clone size or the number of clones.

Our aim was to set the FISH combination of del(17p13), t(4;14), 1q21 gain and del(1p32), four adverse cytogenetic factors rarely evaluated together. In a prospective study of 233 newly diagnosed MM, we observed that the proportion of patients with unfavourable cytogenetic markers could be extended, and that a high level of clonal heterogeneity could be identified by a sufficient number of FISH probes.

## Methods

### Patient samples

Between January 2013 and August 2015, BM samples were collected from 233 patients during diagnosis at the Cytogenetic Laboratories in Valenciennes General Hospital, and Versailles General Hospital. Patients were involved on the basis of WHO 2008 diagnostic criteria for MM. The institutional ethics committee approved the study.

### Plasma cell enrichment

PC were enriched from BM mononuclear cells, using a magnetic cell-sorting CD138 MicroBeads kit (Miltenyi Biotec; BergischGlabach, Germany) according to the manufacturer’s protocol. BM samples were filtered with a 70 μm filter (Miltenyi Biotec), and suspended in RPMI solution (Dutscher; Brumath, France). After centrifugation (160 G), and removal of the supernatant, mononuclear cells were marked with CD138 microbeads at 4 °C for 15 min. The cells were then separated on a column kit separator (Miltenyi Biotec). PC enrichment was controlled on a cytospin slide by a cytologist. The median efficiency of CD138 selection was 97.5%.

### Interphase fluorescent in situ hybridization

FISH was performed on samples enriched in accordance with the manufacturer’s instructions. The FISH panel included a *TP53/CEP17* probe (Amplitech, Compiègne, France), a *1p32/CDKN2C-FAF1 – 1q21/CKS1B* probe (Amplitech), a t(4;14)(p16;q32) probe (MetaSystems, Altlussheim, Germany), and an *IGH* break-apart probe (MetaSystems).

An automated reading with images captured was performed on GSL-10 Leica (Leica Biosystems; Wetzlar, Germany) and analysed with CytoVision software (Leica Biosystems). The number of PC with abnormal signal patterns was calculated as the average of data from two cytogeneticists who analysed 200 cells. The quality of hybridization was controlled for each FISH technique on metaphases from negative samples.

Technical thresholds were determined for each probe, using isolated CD138-expressing PC from patients without MM, on the basis of the same method as used with patients with MM. Between five and ten PC controls were used for each probe. Thresholds were assessed after counting 200 cells for each negative sample, and established by a “mean + 3 DS” calculation. The technical thresholds for adverse cytogenetic abnormalities were 6.5% for del(1p32), 4.5% for 1q21 gain, 4% for t(4;14) translocation, 4% for *IGH* rearrangement, and 5% for del(17p13).

Compared technical thresholds from the literature were defined as 10% for fusions and 20% for numerical abnormalities [15].

### Statistical analysis

A comparison of numerical variables between two groups was performed using the nonparametric Mann-Whitney *U* test. A comparison of qualitative variables was performed using the Fisher *F*-test. Statistical significance was defined as *p < 0.05.*

## Results

Among the 233 patients, there were 52.3% males (*n* = 122) and 47.7% females (*n* = 111), with a median age of 67 years (41 to 89 years-old). The repartition of ISS score was respectively 37.6%, 33.6% and 28.8% for stage I, stage II, and stage III.

**Fig. 1:**
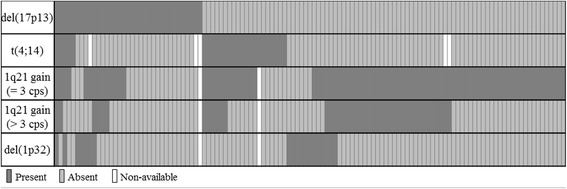
Landscape of the association of adverse abnormalities at diagnosis (*n* = 121). Preferential associations were found between t(4;14) and 1q21 gain (68.0% of cases in t(4;14) subgroup), and between del(17p13) and del(1p32) (36.8% of cases in del(17p13)^+^ subgroup). 1q21 amplification in more than 3 copies was found in 48.3% as 1q21 gain side-line. (cps = copies)

Cytogenetic abnormalities were identified in 79.0% of cases (184/233) by FISH analysis (Table 1), with one or more adverse abnormalities in 51.9% (121/233). We observed a del(17p13) in 15.0%, a t(4;14) translocation in 11.5%, a 1q21 gain in 37.8%, and a del(1p32) in 8.7% of patients (Fig. 1). By adding the 1p32/1q21 FISH probe, we were able to identify one or more adverse abnormalities in 39.0% (64/164) of patients with an absence of *TP53* deletion or t(4;14). For these patients, a 1q21 gain and a monoallelic del(1p32) were found respectively in 90.6% and 18.7% of cases. Both markers were associated in 6 cases.

**Table 1:** Distribution of FISH abnormalities

Cytogenetic abnormalities [number of patients with available data]	n [%]
*1p32/CDKN2C-FAF1 – 1q21/CKS1B* probe [*n* = 230]	
1q21 gain = 3 copies	87 [37.8]
1q21 gain >3 copies	42 [18.3]
1p32 monoallelic deletion	19 [8.3]
1p32 biallelic deletion	1 [0.4]
monosomy 1	4 [1.7]
trisomy 1	7 [3.0]
*TP53/CEP17* probe [*n* = 233]	
17p13 monoallelic deletion	35 [15.0]
monosomy 17	11 [4.7]
trisomy 17	32 [13.7]
trisomy 17 with one *TP53* loss	6 [2.6]
*IGH* break-apart and t(4;14)(p16;q32) - *IGH/FGFR3* probes [*n* = 217]	
total *IGH* rearrangements	75 [34.6]
t(4;14)(p16;q32)	25 [11.5]
monosomy 4 or 4p16 deletion	4 [1.9]
monosomy 14 or 14q32 deletion	12 [5.2]
*IGHv* loss	17 [12.8]
4p16 gain	23 [10.9]
14q32 gain	8 [3.4]

*Abbreviations*: *CDKN2C* Cyclin-Dependent Kinase Inhibitor 2C, *CEP17* Chromosome 17 centromere, *CKS1B* Cyclin-Dependent Kinases Regulatory Subunit 1, *FAF1* FAS-associated Factor 1, *FGFR3* Fibroblast Growth Factor Receptor 3, *FISH* fluorescence in-situ hybridization, *IGH* Immunoglobulin Heavy Locus, *IGHv* Immunoglobulin Heavy Locus variable region, *TP53* Tumour Protein P53

When adverse cytogenetic abnormalities were present, they were isolated in 70.2% (85/121) and associated in 29.8% (36/121) of cases. In our cohort, statistically significant associations were observed between the presence of 1q21 gain and t(4;14), and between the presence of del(1p32) and del(17p13) (Fig. 1). The 1q21 gain was present in 68.0% (17/25) of patients with t(4;14) translocation versus 35.1% (67/191) of patients without t(4;14) (*p = 0.001*). The del(1p32) was found in 36.8% (7/19) of patients with del(17p13) versus 12.8% (27/211) of patients without del(17p13) (*p = 0.01*). A subgroup of 1q21 gain was identified with a number of 1q21 signals greater than 3. Between 4 and 9 signals were observed in 18.3% of cases that could be considered as 1q21 amplifications. In our cohort, this subgroup was always found as a 1q21 gain side-line, emerging in 48.3% of cases. Derivative FISH abnormalities targeting 1p32, 1q21, 4p16, 14q32, 17p13 and D17Z1 *loci* were frequently identified and are listed in Table 1 for information.

The median sizes of identified adverse clones were 50% for del(17p13), 80% for t(4;14), 52% for 1q21 gain and 77.5% for del(1p32). By applying frequently used technical thresholds to our population (10% for fusions and 20% for numerical abnormalities), 40% of del(17p13) detected would have been considered as negative, as well as 24% of 1q21 gain, 16.5% of del(1p32) and only 4% of t(4;14). These under-detected clones were mainly represented by numerical abnormalities. The difference of FISH sensibilities may be explained by the probes’ design with or without internal control.

Clonal heterogeneity with at least 2 related clones was observed in 51.1% (94/184) of cases. The cases with 3 or more related clones represented 13.6% (25/184) of the population. Adverse abnormalities were significantly more frequent when the number of clones was greater than or equal to 2, with a frequency of 85.1% (80/94) against 45.6% (41/90) when 1 single clone was identified (*p < 0.0001*).

**Fig. 2:**
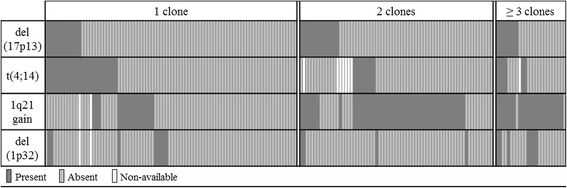
Distribution of abnormalities according to the number of clones at diagnosis (*n* = 184). 1q21 gain was more frequent in the subgroup of 2 clones or more (81.6%) compared to the subgroup of 1 clone (18.4%) (*p < 0.0001*), del(1p32) was more frequent in the subgroup of 3 clones or more (28.0%) compared to the subgroup of 1 or 2 clones (5.8%) (*p = 0.002*). No preferential distribution was observed with del(17p13) and t(4;14) translocation

The distribution of adverse abnormality profiles varies with the number of identified clones (Fig. 2). When clonal heterogeneity was present (≥2 clones), a greater number of MM with 1q21 gain were found: 81.6% (71/94) compared with 18.4% (16/88) when 1 single clone was identified (*p < 0.0001*). In the case of marked clonal heterogeneity (≥3 clones), a higher involvement of del(1p32) was found with a frequency of 28.0% (7/25) against 5.8% (4/69) when only 2 related clones were present (*p = 0.002*). Del(17p13) and t(4;14) were uniformly represented, regardless of the number of clones detected. A higher number of related clones tended to be observed when the ISS stage was III, with a median of 2 clones compared with a median of 1 clone when the ISS stages were I or II (*p = 0.054*).

## Discussion

MM is characterized by the heterogeneity of cytogenetic changes, reflecting the heterogeneity of patients. Cytogenetic analyses have always played a key role in the prognostic evaluation in MM. However, low in-vitro PC proliferation index and low PC infiltration limited the conventional cytogenetic interpretation [5]. Culture failures or misinterpretations of normal karyotypes have been avoided with FISH on CD138-expressing PC [16]. In this study, we investigated 233 MM during diagnosis with a combination of four probes targeting 17p13/*TP53* deletion, t(4;14)(p16;q32) translocation, 1p32/*CDKN2C-FAF1* deletion, and 1q21/*CKS1B* gain.

The prevalence of adverse abnormalities (51.9%) in this cohort of MM at diagnosis is similar to that in previously published data [2, 3, 12, 16–18]. The combination of unfavourable conventionally used cytogenetic markers, del(17p13) and t(4;14) [19], with two more recent markers, del(1p32) and 1q21 gain, has enabled us to identify 39.0% more patients carrying chromosome 1 abnormalities. Indeed, a predominance of 1q21 gain has been observed in 37.8% of cases, as well as a significant number of del(1p32) (8.3%). The need to use a complete panel targeting these four markers in FISH can thus be confirmed.

Preferential associations between adverse abnormalities suggest a successive hits mechanism by analogy with the oncogene involvement in high-grade lymphomas. Several studies assess or stratify clinical risk by combining cytogenetic abnormalities with biomarkers. Two studies mention the accumulation of anomalies in MM with a score test based on the number of anomalies detected, combined with ISS stage [20, 21]. We observed that the accumulation of abnormalities is associated with clonal heterogeneity, considered as unfavourable in most hematologic malignancies. This parameter has not yet been integrated into prognostic models of various studies, even if a recent study indicates that the clone size affects patient outcomes [22]. As noted in previously published data, no association was observed between the number of adverse abnormalities and the ISS stage [11, 13]. Nevertheless, it seems that the clonal heterogeneity increases with the ISS stage III. This would support the idea of including clonal heterogeneity in a revised score.

The preceding remark raises the issue of how the thresholds of FISH defects are determined and used. The guidelines defined thresholds depending on the type of probe or thresholds based on prognostic switches. Such thresholds are either technical with 10% for fusions and 20% for numerical abnormalities [15], or prognostic from 60% up to 70% for del(17p13), or from 10% up to 30% for 1q21 gain and t(4;14) [12, 23–26]. In this study, we used our in-house establishment “mean + 3 standard deviation” from normal isolated PC from BM. Our technical thresholds have enabled us to compare MM data from FISH with the same matrix as that used for the negative control. Thus, we were able to report all anomalies above these thresholds and identify all determinable clones with our four probes. Clonal heterogeneity would either have been less marked with previously published technical thresholds, or smoothed if prognostic thresholds were applied.

Detection of chromosome 1 abnormalities becomes relevant with the emergence of new therapies and therapeutic strategies. Indeed, it could identify 39.0% more patients with valuable prognostic information. Unlike t(4;14) whom negative impact can be erased by proteasome inhibitors [8], the 1q21 gain maintains a lower overall survival rate irrespective of treatment modality [13, 14]. We observed that 1q21 gain was associated with a higher clonal heterogeneity at diagnosis. Moreover, the acquisition of this anomaly is often secondary, especially with t(4;14) translocation. Studies show the lack of 1q21 gain in monoclonal gammapathy of undetermined significance compared to MM, suggesting a significant role of the 1q21 gain in MM progression [27, 28]. These data support the notion of 1q21 gain integration in the current cytogenetic classification in order to define therapeutic cytogenetic profiles, pending the establishment of consensual prognostic profiles.

The deletion of 1p32 *locus* is considered as a strong independent negative prognostic factor [12], and is associated with other adverse abnormalities in 2/3 of cases in our series. Del(1p32) potentiates the unfavourable characters of del(17p13) or t(4;14) translocation [12]. This deletion was identified in the presence of strong clonal heterogeneity (≥3 related clones) reflecting a major chromosomal instability. It should therefore be a cytogenetic target in any FISH panel at MM diagnosis.

Cases with 2 or more trisomies are poorly represented in our series (7.8%) (Table 1), while hyperdiploid MM represent approximately 50–55% of cytogenetic anomalies in MM [29, 30]. This discordance arises because chromosomes 1, 4, 14 and 17 are poor indicators of hyperdiploidy in MM. Chromosomal gains affect preferentially the odd chromosomes 3, 5, 7, 9, 11, 15, 19 and 21 [31]. As a result, no conclusions about MM ploidy can be reached with our probes, unless we add a chromosomal centromeric marker among the most common trisomies, such as chromosomes 5, 9 or 15 in the set of FISH probes sorting [3, 29]. Cases with monosomies were rare, but consistent with published data, considering the non-use, in our cohort, of chromosome 13 probe known as the most frequent monosomy in MM [30].

## Conclusion

The use of FISH on sorted PC has revolutionized the genetic analysis of MM. The absence of chromosomal banding analysis complicates the process of obtaining complete data concerning the combination of cytogenetic markers. However, in this study, we were able to identify adverse abnormalities or derivative anomalies, as well as related clones or clonal evolution by FISH analysis. We also confirm the presence of clonal heterogeneity and accumulation of adverse abnormalities in the first diagnostic analysis. The prognostic impact of these parameters should be evaluated, and could be included in future cytogenetic classifications.
